# Decision Regret after Laparoscopic Sleeve Gastrectomy—5 Years’ Perspective

**DOI:** 10.1007/s11695-021-05480-0

**Published:** 2021-05-25

**Authors:** Katarzyna Bartosiak, Michał R. Janik, Piotr Kowalewski, Maciej Walędziak, Andrzej Kwiatkowski

**Affiliations:** 1 Department of General, Oncological, Metabolic and Thoracic Surgery, Military Institute of Medicine, Warsaw, Poland; 2 Polish School of Bariatric, Warsaw, Poland

**Keywords:** Laparoscopic sleeve gastrectomy, Regret score, Quality of life, Weight loss

## Abstract

**Introduction:**

Patient's satisfaction after weight loss surgery is in the research spotlight. However, there are still no quantitative data regarding whether patients regret their decision to undergo laparoscopic sleeve gastrectomy (SG).

**Objectives:**

The present study aimed to evaluate whether patients regret their decision to undergo SG 5 years after surgery. The secondary objective was to identify whether weight loss and a higher quality of life (QoL) score correlate with the regret expressed by patients.

**Setting:**

Military Hospital, Poland

**Methods:**

A telephone survey was carried out among patients 5 years after surgery. Patient satisfaction regarding their decision to undergo SG was assessed using the Decision Regret Scale. QoL scores were determined using the 36-Item Short Form Survey (SF-36).

**Results:**

One hundred and four patients who answered a full telephone survey were enrolled in the study. Change in body mass index (ΔBMI) was 12.31±6.2, excess body mass index loss (%EBMIL) was 55.45%±25.52%, and percent total weight loss (%TWL) was 25.20%±11.7%. At the 5-year postoperative telephone survey, the mean general health score was 50.96±14.0 and the mean regret score was 32.33±13.24 (range, 25–85). A statistically significant negative correlation was observed between %EBMIL and regret score (*r*=−0.435; *p*<0.001). There was a significant negative association between regret score and energy/fatigue QoL (*r*=−0.205; *p*=0.040). Only eight patients (7.69%) scored >50 on the Decision Regret Scale, which was considered to represent overall regret for their decision.

**Conclusion:**

Our study suggests that, in general, patients did not regret their decision to undergo SG.

**Key Points:**

The majority of patients did not regret their decision to undergo SG.

There was a statistically significant negative correlation between weight loss and patients’ feelings of regret.

Energy/fatigue QoL was the strongest correlate of whether patients regretted their decision to undergo SG.

**Graphical Abstract:**

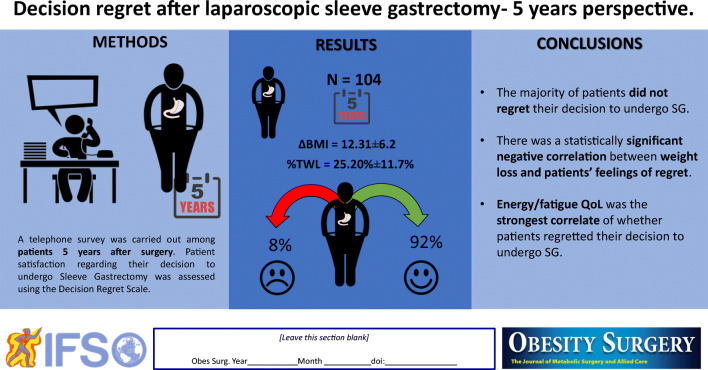

## Introduction

Obesity is a major health problem worldwide. The World Health Organization reported that obesity affected approximately 700 million adults in 2015, with rising trends in most countries [1]. Bariatric surgery is a highly effective treatment option for patients with severe obesity [2, 3].

Numerous studies have reported improvements in quality of life (QoL) after bariatric surgery [4–6]. Although complications after laparoscopic sleeve gastrectomy (SG) are rare [7, 8], they may have a negative effect on patient satisfaction after SG. Moreover, bariatric surgery is associated with short-term perioperative complications and long-term negative effects, including gastrointestinal side effects, malnutrition, and weight gain [9, 10]. Weight-loss outcomes after SG vary significantly, with weight gain occurring over time in many patients [11]. Moreover, patients often have different expectations of weight loss, which results in variation in patient satisfaction after surgical treatment [12]. To date, no quantitative data on whether patients regret their decision to undergo SG have been published.

The present study aimed to evaluate whether patients regret their decision to undergo SG 5 years after surgery. The secondary objective was to identify whether weight loss and a higher quality of life (QoL) score correlate with the regret expressed by patients.

## Methods

### Study Design

The medical records of patients who underwent SG between 2011 and 2014 at a single bariatric center, which performs more than 300 laparoscopic bariatric procedures annually, were analyzed. Patients qualified for surgery if they fulfilled standard criteria established in 1991 by the National Institutes of Health [13].

A telephone survey was carried out among all patients 5 years after surgery. If patients were not contactable after three attempts during the week, contact details were concluded to be outdated and these patients were excluded from the study. The investigator collected information about patients’ demographics, weight loss, comorbidities, and QoL. The study was approved by the institutional bioethical commission (10/WIM/2020).

### Instruments

Patient satisfaction regarding their decision to undergo SG was assessed using the Decision Regret Scale, which was developed by Brehaut et al. [14] to portray decision-making about hormone replacement therapy. A modified version of the scale, which was previously used in bariatric surgery by Wee et al. [15], was used in our study. Patients were asked five questions (Table 1) to assess their feelings about undergoing surgery. Questions 2 and 4 were reverse coded. The scale ranged from 0 to 100, and higher scores reflected patients’ regret about undergoing surgery. A score of >50 demonstrated overall regret for their decision to undergo SG.

**Table 1:** Patients responses on LSG over 5 years

	*n*=101 (%)
Definitely made the right decision	
Definitely yes	84 (83.2%)
Probably yes	10 (9.9%)
Probably no	4 (3.9%)
Definitely no	3 (3.0%)
Regret choice	
Very much	6 (5.9%)
Somewhat	5 (5.0%)
A little	4 (3.9%)
Not at all	85 (84.2%)
Would do it again	
Very likely	81 (80.2%)
Somewhat likely	9 (8.9%)
Not very likely	2 (2.0%)
Not at all	9 (8.9%)
LSG caused negative effects	
A lot	2 (2.0%)
Some	5 (5.0%)
A little	19 (18.8%)
Not at all	75 (74.3%)
How wise was decision	
Very wise	91 (90.1%)
Somewhat wise	6 (5.9%)
Not very wise	4 (3.9%)
Not wise at all	0 (0%)

QoL scores were determined using the 36-Item Short Form Survey (SF-36) [16], a standardized and validated questionnaire used to measure health-related QoL in eight aspects: physical functioning, role limitations due to physical health, role limitations due to emotional health, energy/fatigue, emotional wellbeing, social functioning, pain, and general health. The SF-36 score ranges from 0 to 100, with higher scores indicating better perceptions of health.

### Data Analysis

Analysis was performed using SAS software, University Edition (SAS Institute, Cary, NC, USA). Normality was checked using the Shapiro–Wilk test. For comparison of continuous variables, the Mann–Whitney *U* test or the unpaired Student’s *t*-test was used. Categorical variables were compared using the *χ*^2^ and Fisher’s exact tests. A correlation analysis was used to investigate the association between weight loss and total score on the Decision Regret Scale. Logistic regression was performed to assess the associations between age and body mass index (BMI) and regret.

Weight loss was expressed according to standards developed by Brethauer et al. [17] as a change in body mass index (ΔBMI), percentage of total weight loss (%TWL), and percentage of excessive BMI loss (%EBMIL). Statistical significance was set at *p* < 0.05.

## Results

A total of 459 patients underwent SG from 2011 to 2014. One hundred and four patients who answered a full telephone survey were enrolled in the study. No patients refused to answer questions or participate in the study. However, we were unable to contact 355 patients owing to outdated contact information. There were two unrelated deaths: one patient died of severe pneumonia and another of human papillomavirus-related cervical cancer.

### Demographic Characteristics

Of the total sample of 104 patients, 48 (46.15%) were female and 56 (53.85%) were male. The mean age of patients was 44.07 ± 10.41 years (range, 19–66 years). The mean preoperative BMI was 48.13 ± 6.92 kg/m^2^. Comorbidities included hypertension in 71 patients (68.3%), type 2 diabetes (T2D) in 43 patients (41.3%), hyperlipidemia in 15 patients (14.4%), obstructive sleep apnea in 11 patients (10.6%), cardiovascular disease in 10 patients (9.6%), degenerative joint disease in 12 patients (11.5%), and hypothyroidism in 15 patients (14.4%). Demographic characteristics are summarized in Table 2.

**Table 2:** Basic characteristics of the group

Basic characteristic	*n=*104 (%) or mean (SD)
Sex, *n* (%)	
Women	48 (46.15%)
Men	56 (53.85%)
Age, years	44.07 (±10.41)
Mean BMI, kg/m^3^	48.13 (± 6.92)
Smoker, *n* (%)	10 (10.78%)
Comorbidities, *n* (%)	
Hypertension	71 (68.3%)
Type 2 diabetes	43 (41.3%)
Dyslipidemia	15 (14.4%)
Obstructive sleep apnea	11 (10.6%)
Cardiovascular disease	10 (9.6%)
Degenerative joint disease	12 (11.5%)
Hypothyroidism	15 (14.4%)

*BMI*, body mass index

### Weight Loss

The mean postoperative BMI was 35.71 ± 7.13 kg/m^2^. ΔBMI was 12.31 ± 6.2, %EBMIL was 55.45% ± 25.52%, and %TWL was 25.20% ± 11.7%. Sixty-six patients (63.5%) achieved a %EBMIL of >50% 5 years after surgery.

### Quality of Life

At the 5-year postoperative telephone survey, the mean general health score was 50.96 ± 14.0. Patients achieved the highest QoL scores in the following domains: social functioning (89.5 ± 19.4), physical functioning (85.1 ± 23.6), and limitations owing to emotional problems (83.8 ± 34.5). Patients scored substantially lower in energy/fatigue (58.8 ± 13.6) and emotional wellbeing (59.8 ± 15.2).

### Regret Scale Analysis

The mean regret score was 32.33 ± 13.24 (range, 25–85).

Table 2 presents patients’ retrospective perspectives of their decision to undergo SG. Among patients who were asked if they felt they had made the right decision, 84 (83.2%) answered positively and only 3 (3.0%) expressed dissatisfaction with their decision. Seventy-five patients (74.3%) did not feel any negative effects caused by surgery. Seven patients (6.9%) reported that surgery caused them negative effects. When asked whether they regretted their choice, 85 patients (84.2%) answered negatively and only 4 (3.9%) felt that their decision to undergo SG was unwise. Moreover, 81 patients (80.2%) claimed that if they were given the choice again, they would be very likely to make the same decision.

Age was negatively correlated with regret score (*r* = −0.009; *p* = 0.932); however, there was no significant difference. A statistically significant negative correlation was observed between %EBMIL and regret score (*r* = −0.435; *p* < 0.001) 5 years after SG. Similarly, a statistically significant negative correlation was observed between %TWL and regret score (*r* = −0.455; *p* < 0.001; Table 3). There was a significant negative association between regret score and energy/fatigue QoL (*r* = −0.205; *p* = 0.040; Table 4).

**Table 3:** Association between weight loss and decision regret

	Weight loss, mean (SD)	Association between decision regret
ΔBMI, kg/m^3^	12.31 (± 6.32)	*r*=-0.418, *p*<0.001
%EBMIL, %	55.45 (± 25.52)	*r*= -0.435, *p*<0,001
%TWL, %	25.20 (± 11.70)	*r*=-0.455, *p*< 0.001

*ΔBMI*, change in body mass index; *%EBMIL*, excess body mass index loss; *%TWL*, percent total weight loss

**Table 4:** Association between Quality of Life and decision regret

	QoL score, mean (SD)	Association between decision regret
Physical functioning	85.10 (±23.6)	r= -0.090; p=0.378
Role limitations due to physical health	78.39 (±39.5)	r=-0.059; p=0.571
Role limitations due to emotional problems	83.84 (±34.5)	r=-0.057; p=0.573
Energy/fatigue	58.76 (±13.6)	r=-0.205; p=0.040
Emotional well-being	59.80 (±15.2)	r=-0.071; p=0.480
Social functioning	89.50 (±19.4)	r=-0.185; p=0.066
Pain	63.89 (±34.5)	r= 0.001; p=0.995
General health	50.96 (±14.0)	r=-0.160; p=0.115

*QoL*, quality of Life

Only eight patients (7.69%) scored >50 on the Decision Regret Scale, which was considered to represent overall regret for their decision. Comparing patients who expressed regret (regret score >50) with those who did not (regret score <50), ΔBMI (3.03 kg/m^2^ vs. 13.03 kg/m^2^, respectively; *p* = 0.0004), %EBMIL (14.93% vs. 58.63%, respectively; *p* = 0.0008), and %TWL (6.59% vs. 26.64%, respectively; *p* = 0.0007) were significantly greater. Among the QoL domains, only the social functioning score was significantly greater in patients who did not express regret (*p* = 0.021; Table 5).

**Table 5:** Association between mean weight loss and improvement of Quality of Life on Decision Regret Score

	Regret score > 50; *n*=8 Mean (SD)	Regret score < 50; *n*=83, mean (SD)	*P* value
ΔBMI, kg/m^3^	3.03 (± 4.38)	13.03 (± 65.88)	P<0.001
%EBMIL, %	14.93 (± 25.01)	58.63 (± 22.81)	P<0.001
%TWL, %	6.59 (± 10.26)	26.64 (± 10.55)	P<0.001
QoL, physical functioning,	73.75 (±32.04)	86.11 (±22.71)	p=0.365
QoL, role limitations due to physical health	71.88 (±45.19)	78.98 (±39.19)	p=0.448
QoL, role limitations due to emotional problems	87.50 (±35.36)	83.52 (±34.56)	p=0.635
QoL, energy/fatigue	50.0 (±13.63)	59.52 (±13.37)	p=0.079
QoL, emotional well-being	54.0 (±13.18)	60.30 (±15.35)	p=0.162
QoL, social functioning	81.25 (±14.94)	90.22 (±19.60)	p=0.021
QoL, pain	63.75 (±34.90)	63.90 (±34.70)	p=0.954
QoL, general health	43.13 (19.63)	51.65 (±13.36)	p=0.192

*ΔBMI*, change in body mass index; *%EBMIL*, excess body mass index loss; *%TWL*, percent total weight loss; *QoL*, quality of life

## Discussion

The worldwide increase in obesity has led to a concomitant increase in the number of bariatric procedures, of which SG is the most frequently performed worldwide [18].

In most studies that have focused on weight loss, weight stabilization, and improvements in obesity-related comorbidity, whether patients express regret for their decision to undergo SG has remained unclear. This study aimed to assess regret in patients with obesity 5 years after undergoing SG. Moreover, we considered whether weight loss and higher QoL scores correlated with patients’ feelings of regret.

In our study, only eight patients scored >50 on the Decision Regret Scale, which was considered to represent overall regret for their decision to undergo SG. This group was characterized by less weight loss. There was a statistically significant negative correlation between %EBMIL and regret. Among the QoL domains, there was a significant negative association between regret and energy/fatigue QoL, indicating that patients who had more energy and were less tired were less likely to regret their decision to undergo SG.

Previous quantitative research has largely focused on clinical outcomes after bariatric surgery, and there are limited data on patients’ perspectives on SG. A recent systematic review and thematic synthesis highlighted the profound long-term impact of bariatric surgery on many different aspects of people’s lives and the challenges experienced by patients in coming to terms with these changes [19]. The results of our study confirm the complexity of patients’ experiences. Although the majority of patients assessed did not regret their decision, a small proportion reported some degree of negative effects after SG.

Unsurprisingly, our study shows that the degree of weight loss after SG is a major correlate of regret. Previous data [15] have identified weight loss as a factor that significantly affects patient satisfaction after bariatric surgery. Our findings confirm that the decision to undergo surgery was most frequently regretted by patients who had not lost weight or had achieved a worse outcome than anticipated.

Our research shows that among the QoL domains, there was an association only between regret and energy/fatigue QoL. The results show an improvement in QoL after bariatric surgery. These findings are consistent with those of previous studies [20–23].

Despite patients’ experiences after SG remaining unexplored, recent studies have examined patient satisfaction after Roux-en-Y gastric bypass (RYGB). Turrentine et al. [24] reported that 99% of participants rated their satisfaction with RYGB as high (mean score 8.4, with a score of 10 being highly satisfied). Consistent post-RYGB findings were presented by Wee et al. [15]. Using the Decision Regret Scale, these authors quantified patients’ levels of regret after both RYGB and gastric banding. They reported that up to 20% of patients who had undergone gastric banding expressed regret at having undergone the procedure, whereas approximately 4–8% of patients who had undergone RYGB scored >50 on the Decision Regret Scale (i.e., overall regret with their decision). Our data correspond to research examining regret after RYGB, consistent with a general trend whereby weight loss has the greatest impact on patients’ expressed regret after bariatric surgery.

## Limitations

The present study has several limitations related to its retrospective design and small sample size. Most notably, the limited sample size reduces the accuracy of the weight loss and QoL assessments. Another limitation is the risk of selection bias attributable to our inability to contact each patient who underwent SG. Additionally, the follow-up survey was performed via telephone, which may have been affected by recall bias. A third limitation is the large number of patients lost to follow-up, which resulted from our inability to contact patients owing to outdated phone numbers. Although complications after SG were not included and analyzed in this study, our intention in the next stage is to evaluate the regret score in patients with postoperative complications.

## Conclusion

Our study suggests that, in general, patients did not regret their decision to undergo SG. There was a statistically significant negative correlation between weight loss and patients’ feelings of regret. Energy/fatigue QoL was the strongest correlate of whether patients regretted their decision to undergo SG.
